# Detection of Total Phenol in Green and Black Teas by Flow Injection System and Unmodified Screen Printed Electrode

**DOI:** 10.1155/2010/143714

**Published:** 2011-03-03

**Authors:** Ivanildo Luiz de Mattos, José Heraclito Zagal

**Affiliations:** Departamento de Química de Los Materiales, Facultad de Química y Biología, Universidad de Santiago de Chile, Casilla 40, Sucursal Matucana, Santiago 9170022, Chile

## Abstract

A flow injection system using an unmodified gold screen-printed electrode was employed for total phenol determination in black and green teas. In order to avoid passivation of the electrode surface due to the redox reaction, preoxidation of the sample was realized by hexacyanoferrate(III) followed by addition of an EDTA solution. The complex formed in the presence of EDTA minimizes or avoids polymerization of the oxidized phenols. The previously filtered tea sample and hexacyanoferrate(III) reagent were introduced simultaneously into two-carrier streams producing two reproducible zones. At confluence point, the pre-oxidation of the phenolic compounds occurs while this zone flows through the coiled reactor and receives the EDTA solution before phenol detection. The consumption of ferricyanide was monitorized at 360 mV versus Ag/AgCl and reflected the total amount of phenolic compounds present in the sample. Results were reported as gallic acid equivalents (GAEs). The proposed system is robust, versatile, environmentally-friendly (since the reactive is used only in the presence of the sample), and allows the analysis of about 35–40 samples per hour with detection limit = 1 mg/L without the necessity for surface cleaning after each measurement. Precise results are in agreement with those obtained by the Folin-Ciocalteu method.

## 1. Introduction

Phenolic compounds are a class of chemicals that have a hydroxyl functional group attached to an aromatic hydrocarbon group. The simplest of the class is phenol (C_6_H_5_OH) [1]. The term “polyphenols” refers to a group of chemical substances found in plants which are characterized by the presence of more than one phenol unit, for example, hydrolysable tannins (gallic acid) and phenylpropanoids (flavonoid, lignins, and condensed tannins) [1, 2]. The largest and most studied polyphenols are the flavonoids, as catechins, which correspond to the main phenolic compound found in green and black teas [3]. Gallic acid was identified to be main free phenolic acids; the four major catechins are (−)-epicatechin gallate (ECG), (−)-epigallocatechin (EGC), (−)-epigallocatechin gallate (EGCG), and (−)-epicatechin (EC). These species can be associated to a reduction in the risk of cardiovascular disease and some forms of cancer, as well as the improvement of oral health and other physiological functions such as anti-hypertensive effect, body weight control, antibacterial and antivirasic activity, and so forth [1–4]. Therefore, the development of analytical methods for polyphenols represents an important and exciting topic for analytical chemists. 

For total and/or particular phenol analysis, numerous methods have been reported based, for example, on spectroscopy [5, 6], chemiluminescence [7], spectrophotometry [8–10], chromatography [3, 11], electrochemistry [12–16], and so forth. In spite of the fact that many methods are available, most of them lack versatility, simplicity, and suitability for large-scale analyses. When a rapid determination of “total phenolic compounds” is needed, simple procedures involving an unselective reaction are preferable, especially when the automation of the analytical procedure has proved to be a versatile approach and relevant for quality control [17]. In this context, flow injection system has been widely accepted and the incorporation of the screen-printed electrodes can increase analytical applications because of their characteristics such as small size, planar geometry, flexible construction, lost cost, disposable, and so forth [18].

The preoxidation of phenol by hexacyanoferrate(III) has often been exploited in continuous-flow procedures [19, 20] but it has been shown that it presents a drawback: electrode fouling with the formation of electropolymerized films on the electrode surface, which affects the quality of the analysis. In order to minimize this problem, the use of EDTA has been proposed [21] and can represent an interesting strategy for sensor development. 

With these facts in mind, the main purpose of this work is to develop a fast and low-cost automated procedure for total phenolic compound determination in tea samples by using hexacyanoferrate(III) and EDTA. The preoxidation by ferricyanide and the use of EDTA prevent electrode fouling. The design of the FI system, using the simultaneous introduction of reactive and sample, and the use of unmodified gold screen-printed electrodes contribute to a low-cost approach which is suitable for industrial applications.

## 2. Experimental

### 2.1. Apparatus

**Figure 1:**
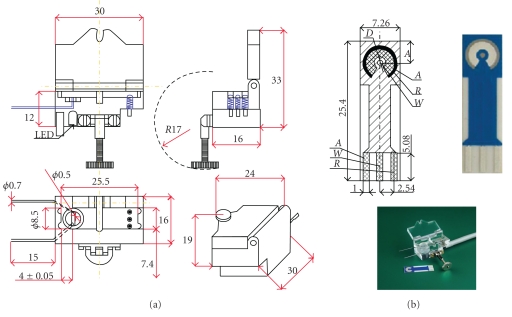
Flow-cell (model FC_2_) and screen-printed electrode developed by BVT Technologies (Czech Republic). *A*, *R*, and *W* refer to auxiliary, reference, and working electrodes. In the present flow-cell does not exist the light emitting diode (LED) as specified in the figure.

Cyclic voltammetry was performed using a PS potentiostat system (Palm Instruments BV, The Netherlands) connected to a PC. A three-compartment electrochemical cell containing three electrodes in the form of an electrochemical sensor type AC1.W.R (BVT Technologies, Czech Republic) was used. The sensor was formed on a corundum ceramic base and on this surface, the working, the reference, and the auxiliary electrodes were placed as illustrated in Figure 1. Its compositions were: AuPd (98/2), Ag/AgCl (60/40), and AuPd (98/2%), respectively. 

Hydrodynamic experiments were carried with the PS potentiostat at amperometric detection technique (*E*_appl._ = 360 mV, Interval = 0.05 s and time run between 1000 r 3000 s). Flow-injection experiments were carried out in an ALITEA eight channels peristaltic pump (Sweden) furnished with a Tygon, injection commutator tubing (0.8 mM, i.d., wall thickness < 0.2 mM) and other accessories. The flow-cell (Figure 1), which ensured the wall-jet flow around the working electrode (model FC_2_), was fabricated by BVT Technologies (Czech Republic).

### 2.2. Reagents, Standards, and Samples

All solutions used were prepared with analytical-grade chemicals such as Folin Ciocalteau reagent, gallic acid monohydrate (3,4,5-trihydroxybenzoic acid), (−)-epicatechin gallate (ECG), (−)-epigallocatechin (EGC), (−)-epigallocatechin gallate (EGCG), (−)-epicatechin (EC), sodium carbonate, potassium hexacyanoferrate, potassium nitrate, and ethylenediaminetetraacetic acid disodium salt dehydrate (EDTA). 

Working standard solutions of gallic acid, ECG, EGC, EGCG, and EC were freshly prepared by dilution of the stock solution with 0.05 M phosphate buffer. The stock solution of gallic acid was prepared by dissolving 0.500 g of dry gallic acid in 10 mL of ethanol and dilute to 100 mL volumetric flask with double distilled water (Millipore-Q). When not in use, it was stored under refrigerator for no more than two weeks. 

Black and green tea samples were provided by local stores. The bags of different tea samples (2 g each) were introduced in a 250 mL erlenmeyer of boiling water and allowed to cool down to room temperature (25°C). After that, the sample solution was filtered using a standard filter paper (Whatman, qualitative). Before injection in the FI system, the tea samples, if necessary, were diluted with the carrier solution for fitting better the calibration curve.

### 2.3. Electrodes

Gold screen printed electrodes were used as received. Initially, they were used for cyclic voltammetry experiments and after that they were placed into the flow-electrochemical cell (Figure 1) for flow amperometric experiments. 

## 3. Results and Discussion

The composition of gallic acid and tea catechins in commercial teas varies with species, season, and horticultural conditions and particularly with degree of fermentation during the manufacturing process. Also, the procedure used for obtaining the samples, for example, the time of boiling water, the use of filtration, type of filter, and so forth. can alters the final results. Therefore, these steps must be well defined. 

For comparative studies, the Folin-Ciocalteau method was used [8]. It is a sensible method for total phenols, and the hydroxyl groups control the color developed which is monitored at 765 nm. Ascorbate is a potential interferent, and reducing sugars (glucose and fructose) can cause minor interferences and must be corrected. Concerning the procedure, the following method was used: (i) 20 *μ*L of blank, standard or sample (ii) 1500 *μ*L of water (iii) 100 *μ*L of the Folin-Ciocalteu reagent (iv) mix the solution and after few minutes, add 300 *μ*L of the sodium carbonate solution (v) left at 40°C for 30 minutes and (vi) determine the absorbance of each solution. The results are reported as gallic acid equivalent (GAE). The total content of phenolic compounds can be calculated according to the formula *C* = (*c* · *V*)/*m*, where c is the concentration of gallic acid established from the calibration curve (mg·L^−1^), *V* is the volume of tea solution (mL), and *m* is the weight of tea (g). This method is not specific and refers to an estimative of total polyphenols contents; however, it is the most used method for this kind of analysis and refers to a international standard method. Results were expressed as galic acid equivalent (GAE) because the phenols in tea contain mostly other phenols and only small amount of gallic acid. The results could be expressed also as catechin gallate because of the low concentration in tea. The phenols found in tea is mainly because the presence of caffeine and catechin as ECG, EGC, EGCG, and EC [3]. 

**Figure 2:**
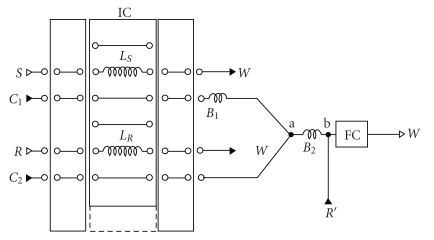
Flow diagram used. *S*, sample (standard solutions or samples), *C*_1_ and *C*_2_, carrier streams (phosphate buffer solution), *R*, reagent stream (ferricyanide solution), IC, injector, *L*_*S*_ and *L*_*R*_, sample and reagent loops, *W*, waste, *B*_1_ and *B*_2_, coiled reactors, a and b, confluent points, *R*′, confluent stream (EDTA solution), FC, flow cell (model FC_2_), and *W*, waste. The boxed part relates to the movable bar of the commutator, the dashed lines indicating the next commutating state. The sites where pumping is applied are indicated by arrows. For system dimensioning, see text.

The preoxidation of gallic acid by ferricyanide and the employment of EDTA were put here as strategic to prevent the passivation of the working electrode during the oxidation of phenolic compound. The proposed FI system represents an interesting alternative for developing robust systems for routine analysis. Before using the flow injection system of Figure 2, gallic acid, ferricyanide, and EDTA solutions were observed by cyclic voltammetry. 

### 3.1. Cyclic Voltammograms of the Gallic Acid and Ferricyanide. Effect of EDTA

**Figure 3:**
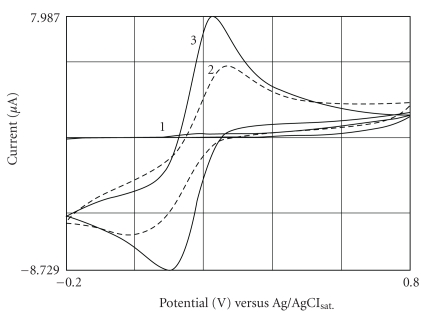
Cyclic voltammograms obtained for 10 mM K_3_Fe(CN)_6_ in 1.0 M KNO_3_ (3) and in presence of 1 mM gallic acid (2). The lower register (1) refers to 1.0 mM gallic acid in 1.0 M KNO_3_. Scan rate = 50 mV s^−1^.

Figure 3 shows cyclic voltammograms of the gallic acid alone (1), ferricyanide alone (3), and ferricyanide plus gallic acid (2). All in presence of 1 M KNO_3_. Profile no. 2 shows the anodic and cathodic peaks at 270 and −17 mV with a formal potential *≈* 143 mV and a peak separation of *ca.* 253 mV. If compared with the profile no. 1 or gallic acid alone, the anodic peak is shifted to more positive potential as a result of a catalytic effect for the oxidation process. Thus, the ferricyanide(III) can be use for oxidizing the gallic acid and the ferrocyanide(II) produced (or residual amount of ferricyanide) can be used as an indicative of the process and monitoring of the gallic acid. The drawback of this monitoring is the passivation of the electrode which requires the cleaning of the electrode after each measurement. Certainly, for industrial application, it is not so attractive. The addition of EDTA to the medium produces a significant enhancement in the electrochemical behavior of phenolic compounds, avoiding electrode passivation [21]. 

**Figure 4:**
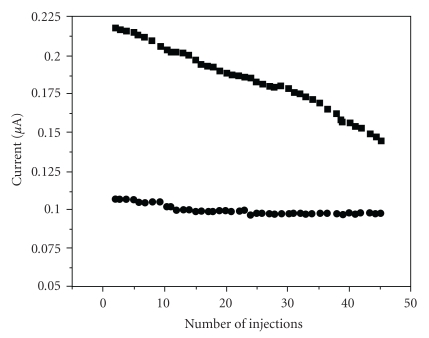
Effect of EDTA solution on the operational stability of the gold screen-printed electrode. High level (100.0 mg·L^−1^, square) or low level (50.0 mg·L^−1^, circle) refers to successive injection of standard-solutions of gallic in phosphate buffer. *R*′ is the flow rate which depending on the experiments; it is distilled water for 100.0 mg·L^−1^ gallic acid and 0.25 M EDTA solution for 50.0 mg·L^−1^ gallic acid.

We believe that the stabilization of the signals occurs because: (i) the oxidation of gallic acid occurs by hexacyanoferrate(III), outside of the electrode, like a preoxidation and (ii) the oxidized gallic acid reacts with EDTA giving a complex GA_ox_ EDTA. The formation of the complex reduces polymerization of the oxidized products formed that passivate the electrode. In fact, Figure 4 shows that in presence of EDTA solution the signals associated to gallic acid was maintained constant after 50 successive injection; in its absence, this signal was deteriorated. Similar results were showed before in experiments using dopamine, catechol, and 4-aminophenol oxidation [21]. Therefore, the preoxidation and the formation of a complex with EDTA can be used to avoid the formation of a passivation layer on the electrode surface and allows the determination of phenolic compounds without the necessity of cleaning the electrode by polishing before each measurement. 

The CV scanned between 800 and −200 mV versus Ag/AgCl with 10 mM hexacyanoferrate(III) plus 1.0 M KNO_3_ (profile no. 3) presented clear anodic and cathodic peaks (3, Figure 3). These peaks were attributed to the reversible Fe(CN)_6_^3−^/Fe(CN)_6_^4−^ redox couple with a peak separation of ca. 130 mV. It is important to clarify that the deviation from the theoretical values [22] is attributed to the ink composition and mainly to the presence of a polymeric binder that affects the redox activity [23]. It is possible that the binder could cover some of the active sites of the electrode surface.

### 3.2. The Flow-Injection System

The flow-injection system is illustrated in Figure 2 and involves two-carrier streams. In the position specified, the sample (*S*) and the reagent (*R*) were aspirated through the *L*_*S*_ and *L*_*R*_ loops, respectively, which defined the injected volumes. When the injector commutator was switched, the loops were placed into the corresponding *C*_1_ and *C*_2_ water carrier streams, producing two reproducible zones which were pushed towards a confluence point. The zone associated with *L*_*R*_ is the first to arrive in order to keep the oxidative ambient at the confluence point; the sample zone (associated with *L*_*S*_) arrives later because it flows through a different analytical path after injection (*B*_1_). At confluence point (a), the total phenol contents (expressed as gallic acid equivalent) are oxidized by hexacyanoferrate(III) while this zone flows through coil *B*_2_. At (b) confluent point, an additional confluent stream of EDTA solution (*R*′ or EDTA reagent) is added in order to avoid fouling of the working electrode. The monitoring of hexacyanoferrate(III) consumption was carried out at 360 mV versus Ag/AgCl and the passage of the processed sample through the flow cell produced a transient peak in the baseline, proportional to the total content in the sample. 

In this system, the peak height is the basis of the measurements and it is proportional to the content of phenols. The flow-injection analysis was designed to provide a moderate dispersion. The flow rate of *C*_1_ and *C*_2_ were fixed as 1.2 mL·min^−1^ as a compromise between the sampling rate and the mean available time for the reduction of hexacyanoferrate(III). Path *B*_1_ (15 cm) was needed to increase sample dispersion and to retard the arrival of the sample zone at the confluent point. The *B*_2_ coil reactor was 50 cm long. Both were defined after preliminary experiments and could be both reduced or increased depending on the kind of sample to be analyzed and the condition of the mixture. The *L*_*S*_ and *L*_*R*_ sampling loops were usually 25 cm long (*ca*. 100 *μ*L). The sample aspiration rate was chosen as 1.2 mL·min^−1^ to simplify the system (all stream with same value) and fill the sampling loop under good conditions. For routine work, the lengths of the sampling and reagent loops could be adjusted to guarantee better results in terms of sample dispersion regardless of the travel analytical path and amount of reagent. This procedure can be conducted employing a standard solution. 

In order to define the composition of the reagent, the potassium ferricyanide concentration was varied between 0.5 and 5.0 mM. The concentrations of solutions of EDTA were varied using concentrations of 0.05, 0.1, 0.2, and 0.5 M. Sample and reactive loops were varied using lengths of 5, 15, 25, and 30 cm. The coiled reactors dimensions were varied as 5, 15, or 30 for *B*_1_ and 25, 50, or 100 cm long for *B*_2_. The speed of the peristaltic pump was investigated in order to know the effect of the mean available times for oxidation of gallic acid. Thus, the flow rates verified were 0.4, 0.8, 1.2, or 1.6 mL·min^−1^, respectively. 

The system shown in Figure 2 with 1.0 mM K_3_Fe(CN)_6_ as (*R*), 0.05 M K_2_HPO_4_/KH_2_PO_4_ as (*C*_1_ and *C*_2_), and 0.25 M EDTA as (*R*′) was used for analysis of the samples. Gallic acid standard (1.0 to 10.0 or 5.0 to 50.0 mg·L^−1^) solutions were used before the analysis to evaluate the flow system and create the analytical curve. Precision was evaluated by calculating the relative standard deviation of the results of eleven successive measurements of a typical tea sample with a gallic content of 39.23 mg·L^−1^. The results obtained from the Folin-Ciocalteu method (six samples) were compared with those obtained by the proposed approach. 

Optimization of the variables involved in the system design were performed by the univariate method.  Table 1 gives the range over which each variable was studied and also the selected values. For these investigations, the flow system shown in Figure 2 was employed with a 25.0 or 50.0 mg·L^−1^of gallic acid standard solution. The potential applied to the working electrode was 360 mV versus Ag/AgCl because for higher (up to 600 mV) or lower (up to −100 mV) the signals presented instability. Increasing the injected volume (25 to 100 *μ*L) led to a favorable effect on the analytical signal. For values greater than 100 *μ*L, the signal did not increase, probably due to the saturation of the electrode surface by adsorbed species. In addition, pronounced losses in the linearity and sampling rate were observed. For 60 *μ*L, good correlations between the current and the concentration, combined with a suitable sampling frequency. Thus, this volume was selected for both sample and reagent.

The influence of flow rate on the analytical signal (not shown) was investigated by varying it from 0.4 to 1.6 mL·min^−1^. For flow rates lower than 0.8 mL·min^−1^, the recorded peak heights were increased as a result of the longer oxidation time of gallic acid by hexacyanoferrate(III). However, the strategy resulted in an unacceptable low sampling rate. For values higher than 1.2 mL·min^−1^, in spite of the high sampling frequency, the system stability decreased. Then, the flow rate was selected as 1.2 mL·min^−1^ for subsequent experiments as a compromise between sensitivity and analytical frequency. 

The effect of the reagent concentration was studied by varying the hexacyanoferrate(III) from 0.5 to 5.0 mM. By increasing the composition of the ferricyanide, reagent was possible to improve the amperometric response of the system but the linear range of response decreased. A concentration of K_3_Fe(CN)_6_  <  0.5 mM, resulted in a loss of linearity, and for higher concentrations, up to 5.0 mM, a good calibration curve was obtained in the range from 100.0 up to 500.0 mg·L^−1^. For higher intervals, poor linearity was observed. We have noted that by changing the ferricyanide concentration it is possible to obtain different analytical curves.  Table 2 shows the performance of different analytical curves obtained by gallic acid (two intervals) and catechin solutions: (−)-epicatechin gallate (ECG), (−)-epigallocatechin (EGC), (−)-epigallocatechin gallate (EGCG), and (−)-epicatechin (EC). This variable (ferricyanide concentration) can be changed to adjust the system depending on the sample to be analyzed. 

The beneficial effect of EDTA on the electrochemical redox process was evaluated by flow amperometry (Figure 4). By using the system of Figure 2, solutions of gallic acid were injected with R′ as water or EDTA solution. Fifty successive injections of 100.0 or 50.0 mg·L^−1^ gallic acid were performed as *R*′ = distilled water or *R*′ = 0.10 M solution of EDTA. 

For the first case (injected standard, 50.0 mg·L^−1^ and *R*′ = EDTA), in 10 injections, the current varied around 0.100 *μ*A. When the EDTA solution was replaced by distilled water, the decrease found was higher from around 0.220 to less than 0.150 *μ*A. In order to improve the performance of the sensor concerning the operational stability, the concentration of EDTA was increased up to 0.25 M. Under these circumstances, the loss in analytical signal was between 2.0 and 3.0%. 

The oxidation of phenolic compounds by hexacyanoferrate(III) is not selective, and for the specific compound determination, is necessary previous treatment of the sample. Teas with addition of ascorbic and citric acid (“tea with lemon”, etc.) must be avoided since standards of gallic acid plus ascorbic acid gave results with significant errors. The samples used in this work were selected, and the low-interferent-to-analyte ratio found restricted the number of potential interferents.

### 3.3. Applications

**Figure 5:**
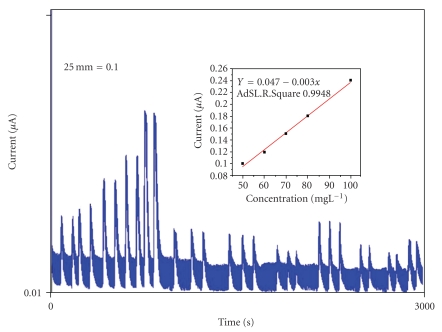
Recorder output of a routine run. From left to right, measurements in duplicate for gallic acid standard solution (50, 60, 70, 80, and 100 mg·L^−1^) followed by signal of phenolic compounds EGCG (50 mg·L^−1^), EC (50 mg·L^−1^), GA (25 and 50 mg·L^−1^), and different samples of tea.

**Figure 6:**
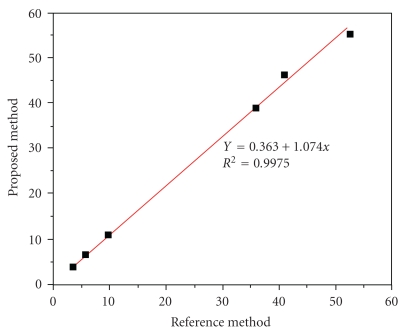
Relationship between proposed and the reference method used for total phenolic compounds detection.

After being dimensioned, the flow system was applied to an analysis of tea. The long-term stability of the system was evaluated by injecting different samples during 4-5 h working periods. For analysis of the black and green teas, the system was adjusted and the calibration curve was obtained in the concentration range from 50.0 to 100.0 mg·L^−1^ of gallic acid. For *n* = 5, the typical regression coefficient was 0.9948 (Figure 5). Using the proposed system, about 35–40 samples can be run per hour with a detection limit of 1.0 mg·L^−1^. The sampling rate can be improved by reducing the mean available time for phenolic compound oxidation. This aspect is particularly important when sensitivity is not critical. For a typical tea sample with a total phenol content of about 40.0 mg·L^−1^, the relative standard deviation of eleven experiments was estimated to be lower than 2.0%. The accuracy was assessed by running six already analyzed samples by the Folin-Ciocalteau method (Figure 6). The linear correlation between both methods was 0.9975.

## 4. Conclusion

The design of an FI system (including the SPE) which introduces simultaneously the reactive and sample for preoxidation and environmentally-friendly analysis is desirable for routine applications. 

The oxidation by hexacyanoferrate(III) is unselective; it means that its use is not appropriate for total phenolic compound monitoring. The use of a low concentration of ferricyanide with the possibility of reagent adjustment, depending on the kind of sample and necessity, results it in an attractive approach. 

The use of EDTA and the preoxidation step are an interesting strategy for avoiding or minimizing passivation of the electrode surface. It is possible to carry out phenol analysis without continuous cleaning of the electrode surface simplifying the setup. The EDTA concentration can be adjusted for different kinds of samples. The use of screen-printed electrodes is attractive, and considering the low cost of the proposed system, it can be used for large-scale analysis and/or for quality control of teas and other beverages. Gold SPE was used for this analysis because it was available at laboratory but the use of carbonaceous SPE could present better results because of less sensitivity to surface oxidation.

## Figures and Tables

**Table 1:** Optimization of variables.

Type	Parameter	Investigated range	Selected value
I	Flow rate/mL·min^−1^	0.4–1.6	1.2
	Path length, *B*_1_/cm	5–30	15
	Reactor, *B*_2_/cm	10–100	50
	Inj. Volume/*μ*L	25–100	60
II	K_3_Fe(CN)_6_/mM	0.5–5.0	1.0
	EDTA/M	0.05–0.5	0.25
III	Potential, mV versus Ag/AgCl	−100 to 600	360

I, II and III, hydrodynamic, chemical, and physical parameters.

**Table 2:** Parameters of regression for phenolic compounds.

Phenolic compounds	Linear range (*μ*g/mL)	Equation	Correlation, *R* ^2^
Gallic acid	5.0–25.0	−0.016 + 0.007*x*	0.9818
	50.0–100.0	−0.047 + 0.003*x*	0.9948
EGCG	12.5–100.0	−0.005 + 0.021*x*	0.9976
EC	12.5–100.0	−0.0002 + 0.023*x*	0.9710
ECG	12.5–100.0	0.003 + 0.022*x*	0.9982
EGC	12.5–100.0	0.008 + 0.022*x*	0.9919

For the interval 5.0–25.0, the concentration of K_3_Fe(CN)_6_ was 1.0 mM; for the others, the concentration of K_3_Fe(CN)_6_ was 2.0 mM.
